# Perioperative Sleep Disturbances and Postoperative Delirium in Adult Patients: A Systematic Review and Meta-Analysis of Clinical Trials

**DOI:** 10.3389/fpsyt.2020.570362

**Published:** 2020-10-14

**Authors:** Hongbai Wang, Liang Zhang, Zhe Zhang, Yinan Li, Qipeng Luo, Su Yuan, Fuxia Yan

**Affiliations:** ^1^Department of Anesthesiology, Chinese Academy of Medical Sciences and Peking Union Medical College, Fuwai Hospital, Beijing, China; ^2^Department of Anesthesiology, Chongqing Traditional Chinese Medicine Hospital, Chongqing, China

**Keywords:** sleep disturbances, surgery, postoperative delirium, adult, meta-analysis

## Abstract

**Background:** The aim of this systematic review and meta-analysis of clinical trials was to investigate the effects of perioperative sleep disturbances on postoperative delirium (POD).

**Methods:** Authors searched for studies (until May 12, 2020) reporting POD in patients with sleep disturbances following the Preferred Reporting Items for Systematic Reviews and Meta-Analyses (PRISMA) guidelines.

**Results:** We identified 29 relevant trials including 55,907 patients. We divided these trials into three groups according to study design: Seven retrospective observational trials, 12 prospective observational trials, and 10 randomized controlled trials. The results demonstrated that perioperative sleep disturbances were significantly associated with POD occurrence in observational groups [retrospective: OR = 0.56, 95% CI: [0.33, 0.93], *I*^2^ = 91%, *p* for effect = 0.03; prospective: OR = 0.27, 95% CI: [0.20, 0.36], *I*^2^ = 25%, *p* for effect < 0.001], but not in the randomized controlled trial group [OR = 0.58, 95% CI: [0.34, 1.01], *I*^2^ = 68%, *p* for effect = 0.05]. Publication bias was assessed using Egger's test. We used a one-by-one literature exclusion method to address high heterogeneity.

**Conclusions:** Perioperative sleep disturbances were potential risk factors for POD in observational trials, but not in randomized controlled trials.

## Introduction

Postoperative delirium (POD) is a state of brain dysfunction following surgery, and it features acute onset and fluctuating occurrence (1). The typical clinical manifestations of POD include alterations of consciousness, attention, and cognition. According to reports, POD affects 11–51% of patients after major surgery, and it is independently associated with prolonged intensive care, long-term postoperative cognitive dysfunction, and increased mortality (2–5). However, the pathogenesis of POD remains unclear, so it is particularly important to identify risk factors to prevent its occurrence.

Perioperative sleep disturbances are common among surgery patients. The disturbances include obstructive sleep apnea (OSA), reduced total sleep time, sleep fragmentation, circadian rhythm disruption, and so on (6–8). Over 40% of patients complained about poor sleep quality during the first night following surgery, and the sleep problems continued several days post-operation (9). Some observational studies reported that patients with poor sleep quality were predisposed to mental disorders including delirium and cognitive dysfunction (10–12). In addition, several randomized controlled trials (RCTs) found that improving sleep quality, be it through medication or other interventions, strikingly decreased the incidence of delirium (13–15). Although multiple studies have supported the viewpoint that sleep problems are significantly associated with delirium, some studies obtained negative results (16–18). Thus, we designed this systematic review and meta-analysis to clarify the effect of sleep disturbance on the incidence of delirium in adult surgery patients.

## Methods

This systematic review and meta-analysis was performed according to the guidelines of the 2009 Preferred Reporting Items for Systematic Reviews and Meta-Analyses (PRISMA) (Supplementary Table 1) (19).

### Search Strategy

Hongbai Wang and Liang Zhang were responsible for document retrieval. We searched the databases of Pubmed, Embase, Cochrane Library, and Web of Science using the PICOS (Population, Intervention, Comparison, Outcome, Study design) method. Our last search was completed on May 12, 2020. The search terms included “sleep” OR “insomnia” OR “sleep disturbance” OR “night” OR “circadian” AND “surgery” OR “operation” OR “postoperative” OR “anesthesia” OR “anesthesia” AND “delirium” OR “confusion” OR “agitation” OR “acute confusional state” OR “acute confusional syndrome,” and the search scope was “title and abstract.” Because we sought to examine all studies about the effect of sleep disturbances on POD incidence in adult patients undergoing surgery, we did not constrain the search terms for study designs.

### Study Selection

Zhe Zhang and Yinan Li performed the screening process for titles and abstracts, while Hongbai Wang and Su Yuan performed the screening process for full texts. The inclusion criteria were (1) participants aged 18 years or older; (2) patients undergoing surgery; and (3) articles reporting the effect of sleep on delirium. The exclusion criteria were: (1) duplicate articles; (2) participants younger than 18 years old; (3) review or meta-analysis; (4) articles published as an abstract, letter, case report, basic research, editorial, note, method, or protocol; (5) articles presented in a non-English language; (6) studies without statistical differences in sleep quality between intervention and control groups; (7) studies without a specific number of patients with sleep problems (observational studies) and/or delirium; and (8) studies including some patients not undergoing surgery.

### Quality Assessment of Included Studies

Qipeng Luo and Su Yuan independently assessed the quality of included studies. For retrospective and prospective observational trials, risk of bias was assessed using the Newcastle–Ottawa Quality Assessment Scale (NOS), which comprises the following three domains: selection, comparability, and outcome for cohort studies (20). There were four stars in the selection domain, two stars in the comparability domain, and three stars in the exposure domain. Trials with seven or more cumulative stars were considered to be of high quality, those with six stars of moderate quality, and those with <6 stars of low quality (20). For RCTs, risk of bias was assessed using the Cochrane Collaboration Risk of Bias Assessment tool, which included the following seven items: random sequence generation, allocation concealment, blinding of participants and personnel, blinding of outcome assessment, incomplete outcome data, selective reporting, and others (bias due to vested financial interest and academic bias). If a trial was found to have one or more of the items associated with high or unclear risk of bias, this trial was classified as high risk (21). If the two authors disagreed on their assessment, they consulted the third or fourth author. Eventually, we reached a consensus.

### Data Extraction

Yinan Li and Qipeng Luo were responsible for extracting the following information: (1) authors; (2) publication year; (3) total number of participants in each study; (4) age range of all the participants; (5) country of publication; (6) percentage of males; (7) procedures that the participants underwent; (8) methods of sleep disturbance assessment; (9) methods of POD assessment; (10) number of patients with and without POD; (11) number of patients with good and poor sleep quality; and (12) the follow-up time. Hongbai Wang and Liang Zhang were responsible for adjusting data discrepancies.

### Outcome Measures

The sole aim of this meta-analysis was to determine whether perioperative sleep disturbances were associated with increased POD in adult surgery patients.

### Data Synthesis

We divided all the included trials into three groups according to their study design to facilitate data synthesis. These were retrospective observational trials (ROTs), prospective observational trials (POTs), and RCTs.

### Data Analysis

RevMan Review Manager version 5.3 (Cochrane collaboration, Oxford, UK) and Stata version 12.0 (Stata Corp, College Station, TX, USA) were used to perform statistical analyses. We assessed the heterogeneity of included studies using the values of *I*^2^ and the Mantel-Haenszel chi-square test (*p-*value for heterogeneity). The values of *I*^2^ <40%, *I*^2^ = 40–60%, and *I*^2^ > 60% indicated low, moderate, and high heterogeneity, respectively (22). If we identified *I*^2^ > 50% or a *p-*value for heterogeneity <0.1, we used a random-effect model to analyze the data. Conversely, if we identified *I*^2^ <50% or a *p-*value for heterogeneity ≥ 0.1, we used a fixed-effect model to analyze the data (23). The dichotomous outcomes were reported as odds ratios (OR) with 95% confidence intervals (CI). Publication bias was assessed using Begg's test, and studies with a *p* < 0.05 were adjusted using trim-and-fill analysis (24). The statistical tests were two-sided, and overall effects with a *p* < 0.05 were considered to exhibit significant differences.

We conducted sensitivity analysis to address high heterogeneity (*I*^2^ > 40%) through the methods of subgroup analysis or one-by-one article removal. We used meta-regression to identify the sources of high heterogeneity according to possible risk factors (25). Meta-regression analyses that produced a risk factor of *p* < 0.05 were followed by subgroup analysis, while those that produced a risk factor of *p* ≥ 0.05 were followed by one-by-one article removal (26).

## Results

### Study Selection

**Figure 1:**
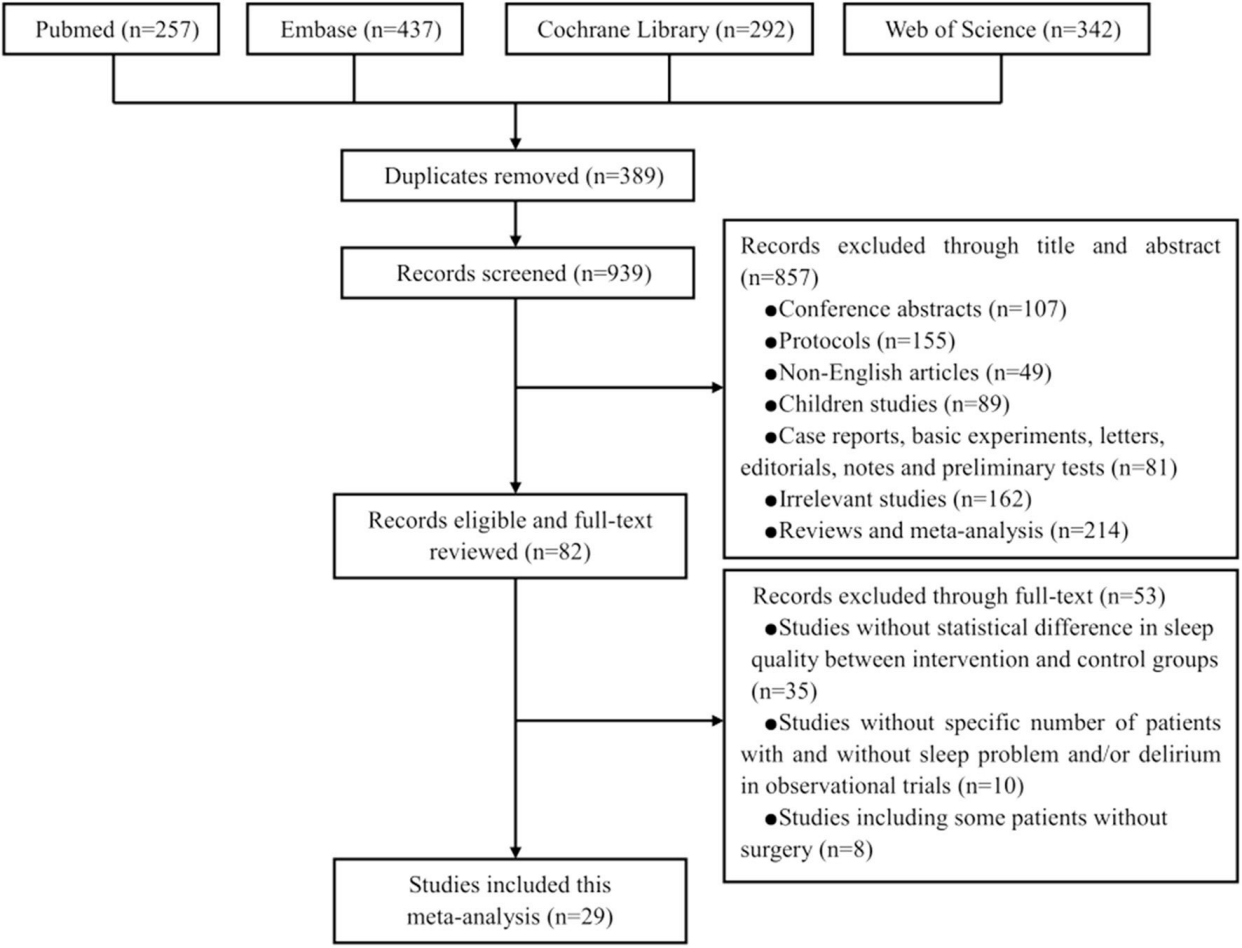
The screening process of the eligible literatures.

Figure 1 presents the PRISMA flow chart for our screening process. We obtained 257 trials from Pubmed, 437 from Embase, 292 from Cochrane Library, and 342 from Web of Science. We removed 389 duplicate trials and excluded 857 trials at the title-and-abstract review stage based on our exclusion criteria. We excluded 53 trials at the full-text review stage, including 35 without statistical differences in sleep quality between intervention and control groups, 10 without a specific number of patients with sleep problems and/or delirium, and eight that enrolled some patients without surgery. Eventually, our search strategy yielded 29 relevant trials with a total of 55,907 patients (Figure 1) (16–18, 27–52).

### Study Characteristics

There were seven trials and 52,369 patients in the ROT group (16, 27–32), 12 trials and 1,435 patients in the POT group (33–44), and 10 trials and 2,103 patients in the RCT group (17, 18, 45–52). Tables 1, 2 presented the basic characteristics of the observational studies (retrospective and prospective) and RCTs, respectively. In one retrospective trial, the control group was matched for age, sex, operated side, type of operation, mode of component fixation, year of operation, surgeon, and type of anesthesia (28). In seven trials, patients only underwent cardiac surgery (33, 36–39, 42, 44), in 20 trials, only non-cardiac surgeries (17, 18, 27–30, 32, 34, 35, 40, 41, 43, 45–52), and in two trials, both cardiac and non-cardiac surgeries (16, 31). All the patients in the included RCTs underwent non-cardiac surgeries. The most prevalent type of non-cardiac surgery was orthopedic surgery. We obtained the mean age of patients in each study by adding the mean age of patients in each group and then dividing by two. In 19 trials, the mean age was 65 years or older (17, 18, 28, 30–32, 34, 35, 37–40, 45, 46, 48–52), though one trial did not provide participants' ages (36). Males accounted for 50% or more of all patients in 16 trials (16–18, 28, 31, 33, 35, 37–39, 41–45, 47), though two trials did not provide sex-related information (36, 51). Three trials did not disclose their method for assessing sleep disturbance (27, 32, 38). Nine trials focused on patients with sleep-disorder breathing or OSA (16, 28, 30, 31, 34, 37, 39, 41, 49), and the other trials studied patients with several sleep problems. Thirteen trials described the effect of preoperative sleep problems on the incidence of POD (16, 27, 28, 30, 31, 33–35, 37, 39–42), and the other trials described postoperative sleep quality.

**Table 1:** The basic characteristics of included observational trials.

**Study**	**Study design**	**No. of patients**	**Country/centers**	**Procedures**	**Age**	**Sleep problem**	**Sleep assessment**
Bosmak et al. (27)	Retrospective	56	Brazil/Single	Knee and hip arthroplasties	18–90 years	Preoperative sleep disorder	NA
Gupta et al. (28)	Retrospective	202 (matched)	USA/Single	Hip or knee replacement	≥18 years	Preoperative OSAS	An RDI of 5 or higher per hour on PSG
He et al. (29)	Retrospective	912	China/Single	MVD procedures	≥18 years	Postoperative sleep disturbance	PSQI>5
King et al. (16)	Retrospective	7,792	USA/Single	major surgery	≥18 years	Preoperative OSAS	A clinician-noted OSA diagnosis or STOP-BANG score>4
Pichler et al. (30)	Retrospective	41,766	USA/Single	Total hip and knee arthroplasties	≥18 years	Preoperative OSA	One or more of the following ICD-9 codes: 786.03, 780.53, 780.51, 780.57, 327.2 X, or 278.03
Strutz et al. (31)	Retrospective	1,441	USA/Single	General anesthesia for a non-neurosurgical inpatient operation	≥18 years	Preoperative OSA	STOP-BANG score
Wang et al. (32)	Retrospective	200	China/Single	Spine, hip replacement, and pelvic or femoral fracture repair	≥65 years	Postoperative sleep disorders	NA
Cheraghi et al. (33)	Prospective	40	Iran/Single	Cardiac surgery	≥18 years	Preoperative Sleep disorder	PSQI > 5
Flink et al. (34)	Prospective	106	USA/Single	Elective knee arthroplasty	≥65 years	Preoperative OSA	PSG
Hwang et al. (35)	Prospective	162	Korea/Single	Surgery due to gastric cancer	≥40 years	Preoperative Sleep disorder	PSQI>8
Koster et al. (36)	Prospective	103	Netherlands/Single	Cardiac surgery	≥45 years	Sleep disturbance after discharge from hospital	Self-report by the patient
Roggenbach et al. (37)	Prospective	92	Germany/Single	Cardiac surgery	>18 years	Preoperative SDB	PSG
Simeone et al. (38)	Prospective	89	Italy/Single	Cardiac surgery	≥18 years	Postoperative sleep disorder (insomnia)	NA
Tafelmeier et al. (39)	Prospective	141	Germany/Single	Cardiac surgery	18–85 years	Preoperative SDB	PSG
Todd et al. (40)	Prospective	101	Germany/Single	Elective hip, knee, or ankle replacement	≥65 years	Preoperative sleep disorder	PSQI>5
Wang et al. (41)	Prospective	128	USA/Single	Major thoracic surgery	≥18 years	Preoperative Intermediate-High Risk for OSA	STOP-BANG questionnaire score ≥3
Wang et al. (42)	Prospective	186	China/Single	Cardiac surgery	≥18 years	Preoperative sleep disorder	PSQI>5
Yamagata et al. (43)	Prospective	38	Japan/Single	Head and neck cancer surgery	33–81 years	Postoperative sleep disorder (minor tranquilizer)	Self-report by the patient
Zhang et al. (44)	Prospective	249	China/Single	Cardiac surgery (CABG)	20–84 years	Postoperative poor sleep quality	Self-report by the patient

*OSA, obstructive sleep apnea; OSAS, Obstructive sleep apnea syndrome; PSQI, Pittsburgh Sleep Quality Index; PSG, polysomnography; RDI, respiratory disturbance index; SDB, sleep-disordered breathing; ICD, International Classification of Diseases; CABG, coronary artery bypass graft; NA, not applicable*.

**Table 2:** The basic characteristics of included RCTs.

**Study**	**No. of patients**	**Country/Center**	**Procedures**	**Age**	**Intervention**	**Control**	**Improvement of sleep**
Aizawa et al. (45)	40	Japan/Single	Resection of gastric or colorectal cancer	70–86 years	DFP	Non-DFP	Intervention group: maintaining nocturnal sleep
Guo et al. (46)	160	China/Single	Tumor resection surgery	65–80 years	Group I: MNI	Group U: usual care	Intervention group: maintain a good sleep-wake cycle
Le Guen et al. (47)	41	France/Single	Major non-cardiac surgery	≥18 years	Routine care plus eye mask and earplugs after surgery	Routine care after surgery	Intervention group: a better sleep quality
Musclow et al. (48)	166	Canada/Single	Total hip replacement and total knee replacement	≥18 years	Usual care + LAO	Usual care	Intervention group: a better sleep quality
Nadler et al. (49)	114	USA/Single	Elective knee or hip arthroplasty	≥50 years	CPAP intervention	Routine care	Intervention group: decreasing incidence of OSA
Potharajaroen et al. (50)	61	Thailand/Single	Surgery	≥50 years	Active intervention: usual care+ BLT	Usual care + a light source of 500 lux.	Intervention group: decreasing incidence of insomnia
Su et al. (51)	700	China/Single	Non-cardiac surgery	≥65 years	Dexmedetomidine 0·1 μg/kg per h within 1 h after ICU admission	Normal saline	Intervention group: improving subjective sleep quality (NRS)
Sultan et al. (52)	203	Egypt/Single	Hip arthroplasty	>65 years	1. Melatonin: 5 mg melatonin at sleep time and another 5 mg 90 min before operative time 2. Midazolam: 7.5 mg midazolam at sleep time and another 7.5 mg 90 min before operative time 3. 100 μg clonidine at sleep time and another 100 μg 90 min before operative time	Nothing as premedication	Intervention group (Melatonin, midazolam, clonidine): significantly increased sedation score
Sun et al. (17)	557	China/Single	Major elective non-cardiac surgery	≥65 years	PCIA: 4.8 μg/kg dexmedetomidine, 2 μg/kg sufentanil and 6 mg tropisetron	PCIA: Normal saline, 2 μg/kg sufentanil and 6 mg tropisetron	Intervention group: improving subjective sleep quality (RCSQ)
Wu et al. (18)	61	China/Single	Non-cardiac surgery under general anesthesia	≥65 years	Dexmedetomidine: 0·1 μg/kg per h for 15 h after surery	Normal saline	Intervention group: prolonged total sleep time, higher Sleep efficiency

*DFP, Delirium-Free Protocol; MNI, multicomponent non-pharcologic interventions; LAO, long-acting opioids; CPAP, continuous positive airway pressure; BLT, bright light therapy; NRS, numerical rating scale; OSA, obstructive sleep apnea; PCIA, patient-controlled intravenous analgesia; RCSQ, Richards Campbell Sleep Questionnaire*.

We divided the patients in each study into two groups according to their sleep quality: one good sleep quality group and one poor sleep quality group (Table 3). The numbers of patients with good and poor sleep quality are shown in Table 3, alongside the numbers of patients with and without POD in each group. The onset time of POD was more than 3 days after surgery in 21 trials (16–18, 27–30, 32, 33, 35–38, 40–45, 48, 51).

**Table 3:** The number of patients with POD under different sleep quality and assessment methods of POD.

**Study**	**Study design**	**No. of patients in each group**		**No. of patients with delirium in each group**		**Observational (or follow-up) time**	**Method of delirium assessment**
		**Good sleep quality**	**Poor sleep quality**	**Good sleep quality**	**Poor sleep quality**		
Bosmak et al. (27)	Retrospective	40	16	4	1	1–4 days after surgery	Diagnoses that included descriptions such as “confused and agitated,” “confused and disoriented,” “confused and drowsy” and “periods of confusion” were considered delirium
Gupta et al. (28)	Retrospective	101	101	3	10	1–5 days after surgery	Noted by caregivers
He et al. (29)	Retrospective	833	79	175	46	From 2–5 days after surgery	DSM-V
King et al. (16)	Retrospective	5,748	2,044	2,740	897	1–7 days after surgery	CAM-ICU
Pichler et al. (30)	Retrospective	38,538	3,228	851	71	NA	ICD-9 codes
Strutz et al. (31)	Retrospective	268	1,173	44	263	1–3 days after surgery	The 3-min diagnostic CAM (3D-CAM) or the CAM-ICU
Wang et al. (32)	Retrospective	102	98	3	14	1 week after surgery	CAM
Cheraghi et al. (33)	Prospective	21	19	1	8	The second to fifth day after the surgery	CAM-ICU
Flink et al. (34)	Prospective	91	15	19	8	Assessments for delirium on postoperative days 2 and 3	The CAM and the DRS-R-98
Hwang et al. (35)	Prospective	141	21	16	3	Before surgery and at 1, 2, 3, and 6–7 days after surgery	DRS-R-98
Koster et al. (36)	Prospective	74	29	10	9	1–1.5 years after surgery	DSM-IV
Roggenbach et al. (37)	Prospective	9	83	3	41	1–4 days after surgery	CAM-ICU
Simeone et al. (38)	Prospective	33	56	5	31	During intensive therapy following cardiac surgery	CAM-ICU
Tafelmeier et al. (39)	Prospective	69	72	11	22	The day of extubation and for a maximum of 3 days	CAM-ICU
Todd et al. (40)	Prospective	43	58	5	22	A minimum of 5 d or until discharge if duration of stay was <5 days	CAM or ICD-10
Wang et al. (41)	Prospective	31	97	6	26	Throughout their entire hospital stay after surgery	CAM-ICU
Wang et al. (42)	Prospective	80	106	6	23	1–7 days after surgery	CAM-ICU
Yamagata et al. (43)	Prospective	25	13	3	7	Between 2 and 5 d after surgery	Diagnosed on the basis of medical records
Zhang et al. (44)	Prospective	192	57	40	36	From the day of surgery to the sixth postoperative day	CAM-ICU
Aizawa et al. (45)	RCT	20	20	1	7	Seven consequent days after surgery	DSM-IV
Guo et al. (46)	RCT	81	79	10	25	1–3 days after surgery	CAM-ICU
Leguen et al. (5)	RCT	20	21	0	3	The first postoperative 24 h	NA
Musclow et al. (48)	RCT	84	82	10	3	Throughout their entire hospital stay after surgery	Neecham Confusion Scale
Nadler et al. (49)	RCT	58	56	12	9	On postoperative day 2	DRS-R-98
Potharajaroen et al. (50)	RCT	30	31	2	11	Within the three days following surgery	CAM-ICU
Su et al. (51)	RCT	350	350	32	79	1–7 days after surgery	CAM-ICU
Sultan et al. (52)	RCT	154	49	46	16	In the three postoperative days	AMT score <8
Sun et al. (17)	RCT	281	276	33	38	The 5 postoperative days	CAM-ICU and CAM
Wu et al. (18)	RCT	31	30	2	2	During the first 7 days after surgery	CAM-ICU

*CAM-ICU, the Confusion Assessment Method for Intensive Care Unit; DSM-IV, the Diagnostic and Statistical Manual of Mental Disorders 4th Edition; DSR-R-98:delirium rating scale-revised-98; ICD, International Classification of Diseases; AMT, Appreviated Mental Test; NA, not applicable*.

### Study Quality

We used NOS to assess the risk of bias in observational studies (retrospective and prospective), and 15 trials obtained seven stars or more, indicating high quality (Supplementary Table 2) (16, 28–32, 34–37, 39–42, 44). We used the Cochrane Collaboration Risk of Bias Assessment tool to assess the risk of bias in RCTs. Many of the included studies demonstrated low risk of bias, as they clearly assessed random sequence generation (seven studies-70%), allocation concealment (eight studies-80%), blinding of participants (seven studies-70%), blinding of outcome assessment (10 studies-100%), incomplete outcome data (nine studies-90%), and selective outcome reporting (nine studies-90%). Six RCTs were found to be high quality (Supplementary Figures 1, 2) (17, 18, 46–48, 51).

### Publication Bias

**Figure 2:**
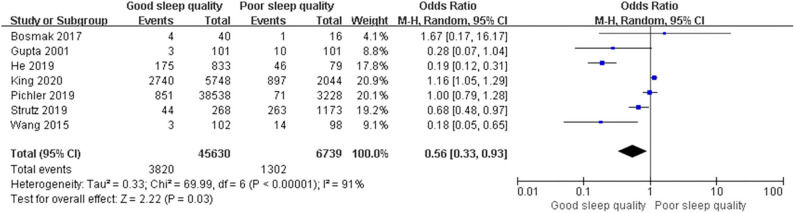
Publication bias of included trials by Egger's test. **(A)** ROTs group; **(B)** POTs group; **(C)** RCTs group.

We assessed publication bias using Egger's test by Stata 12.0 software. We did not find publication bias in the ROT (*p* = 0.085), POT (*p* = 0.764), or RCT (*p* = 0.933) groups (Figure 2 and Supplementary Table 3).

### Post-operative POD

**Figure 3:**
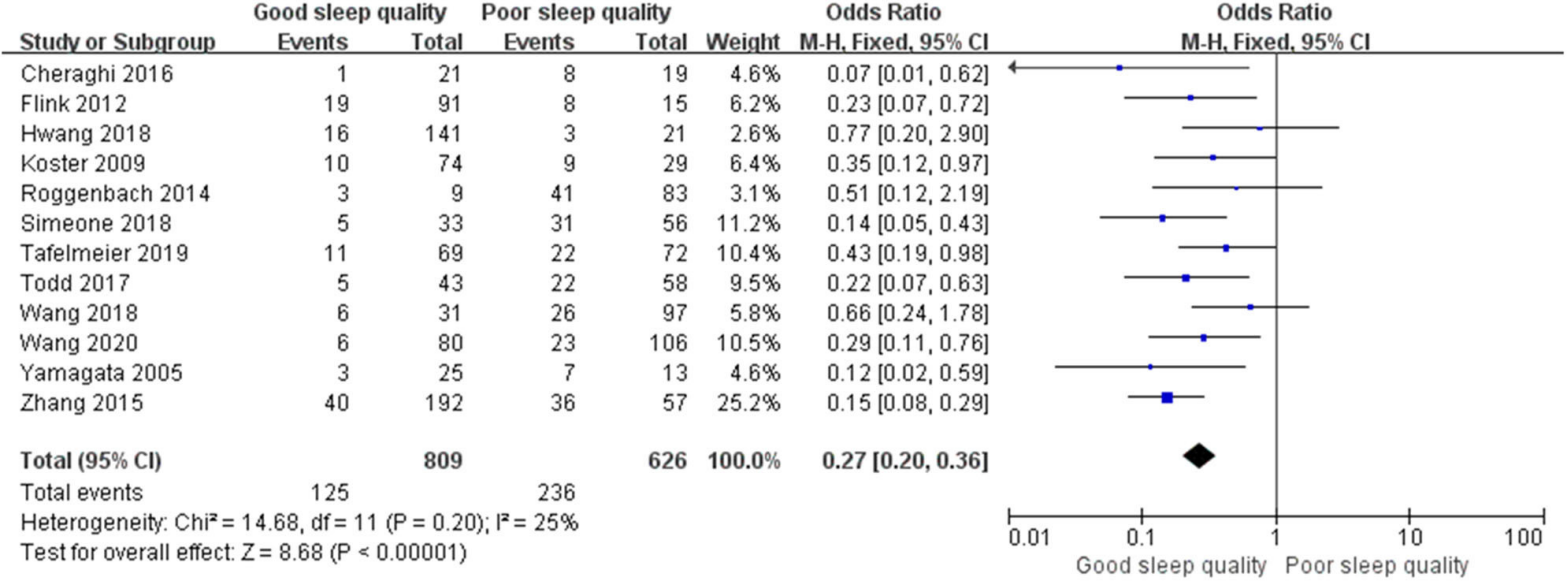
The pooled results of POD incidence after surgery between the patients with good and poor sleep quality in ROTs group.

**Figure 4:**
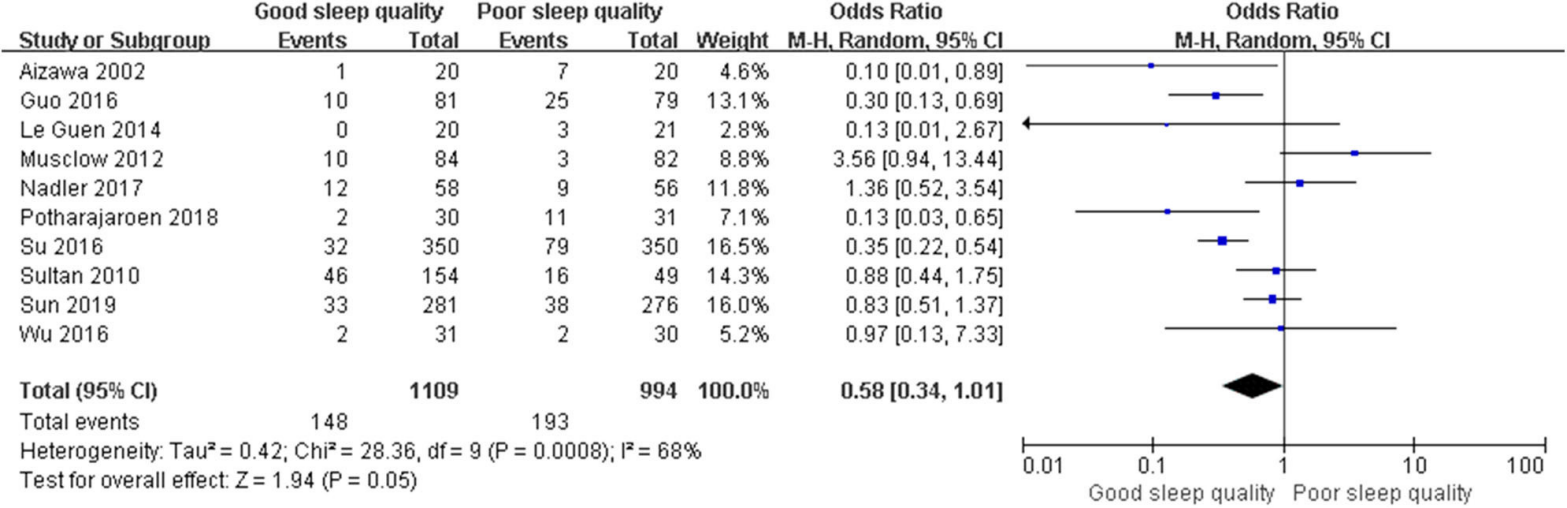
The pooled results of POD incidence after surgery between the patients with good and poor sleep quality in POTs group.

**Figure 5:**
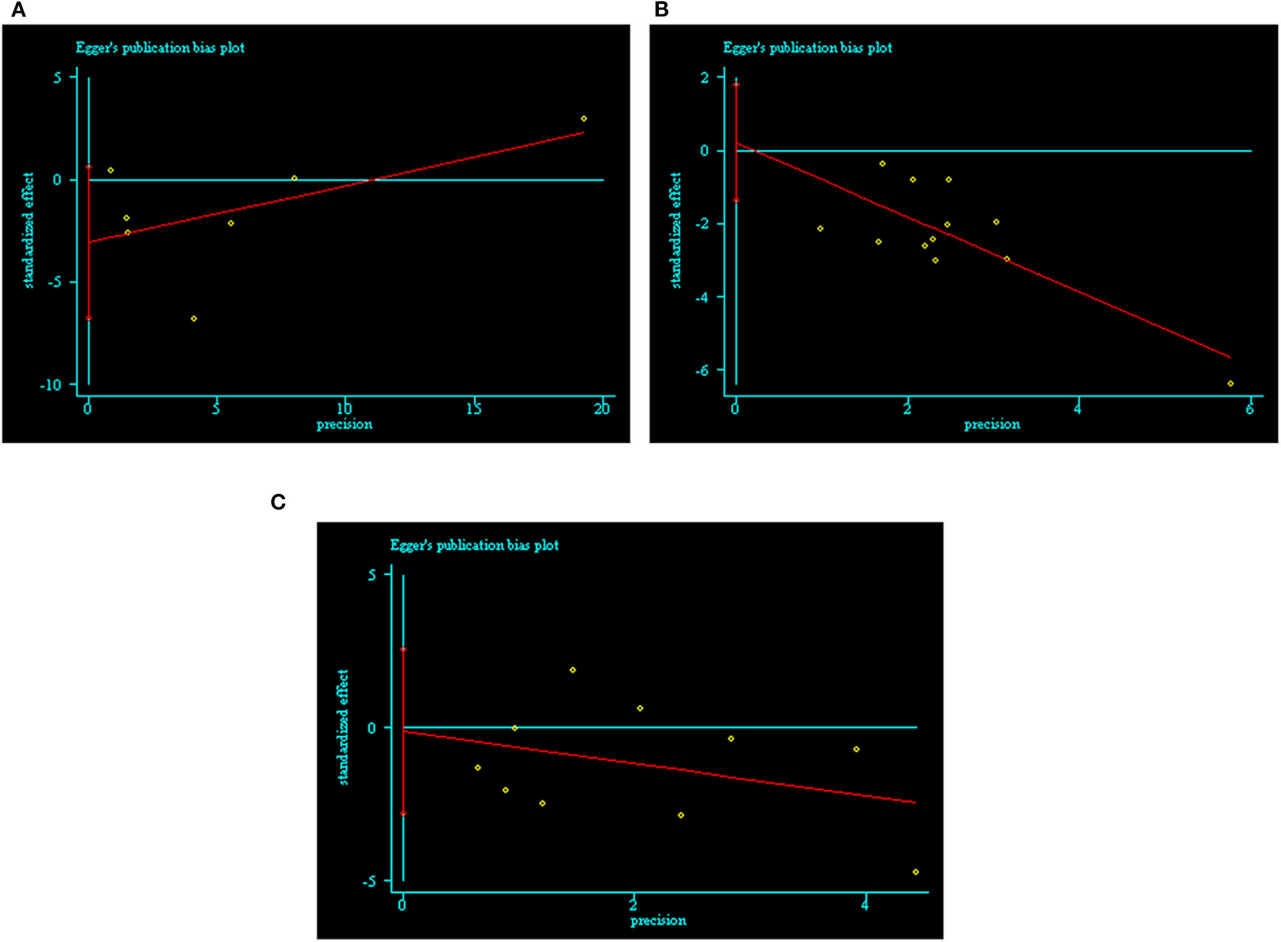
The pooled results of POD incidence after surgery between the patients with good and poor sleep quality in RCTs group.

We used a random-effect model with OR in the ROT (*I*^2^ = 91%) and RCT (*I*^2^ = 68%) groups due to high heterogeneity, and a fixed-effect model with OR in the POT group (*I*^2^ = 25%) due to low heterogeneity. The pooled results of the ROT group (OR = 0.56, 95% CI: [0.33, 0.93], *I*^2^ = 91%, *p* for effect = 0.03) and POT group (OR = 0.27, 95% CI: [0.20, 0.36], *I*^2^ = 25%, *p* for effect <0.001) demonstrated significant differences between patients with good and poor sleep quality in incidence of POD after surgery (Figures 3, 4). However, the pooled results of the RCT group (OR = 0.58, 95% CI: [0.34, 1.01], *I*^2^ = 68%, *p* for effect = 0.05) showed no significant differences between patients with good and poor sleep quality (Figure 5).

### Sensitivity Analysis

We performed meta-regression to identify the sources of heterogeneity in the ROT and RCT groups, assessing possible risk factors including publication year, average age (≥65 years and <65 years), male proportion (≥50% and <50%), surgery types (non-cardiac surgery, cardiac surgery, and cardiac and non-cardiac surgeries), onset time for POD (>3 days and ≤ 3 days), and study quality (low quality and high quality). Unexpectedly, all *p-*values for these risk factors were over 0.05 (Supplementary Tables 4, 5). Afterwards, we used the method of one-by-one literature removal and found that 4 trials were the main sources of heterogeneity in the ROT group (*I*^2^ dropped from 91 to 12%) and four trials were in the RCT group (*I*^2^ dropped from 68% to 19%).

**Figure 6:**
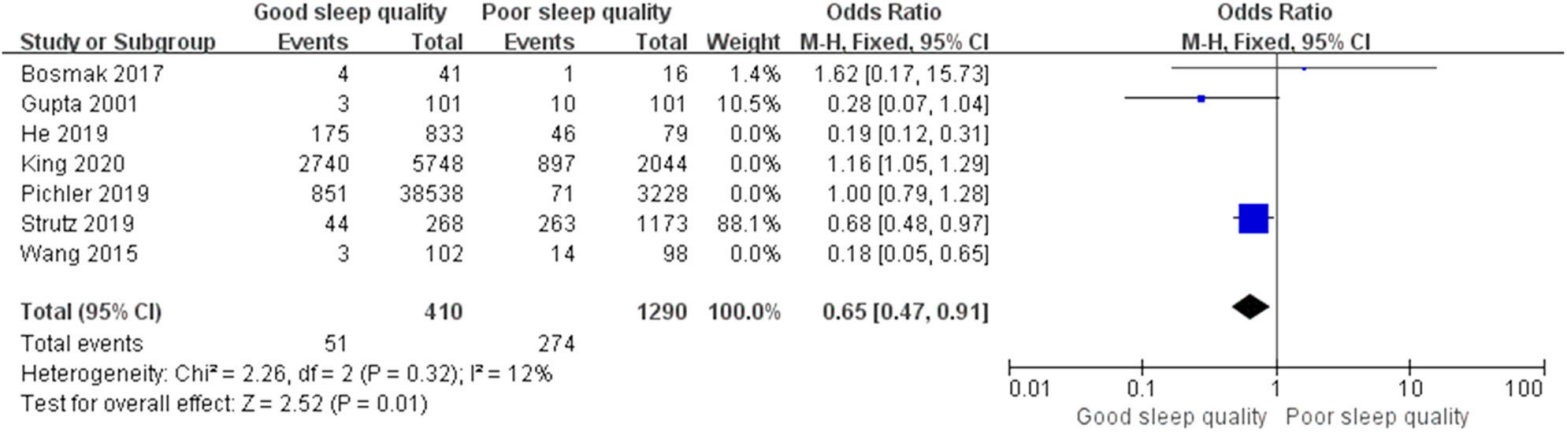
The pooled results of POD incidence after surgery between the patients with good and poor sleep quality in ROTs group after sensitivity analysis.

**Figure 7:**
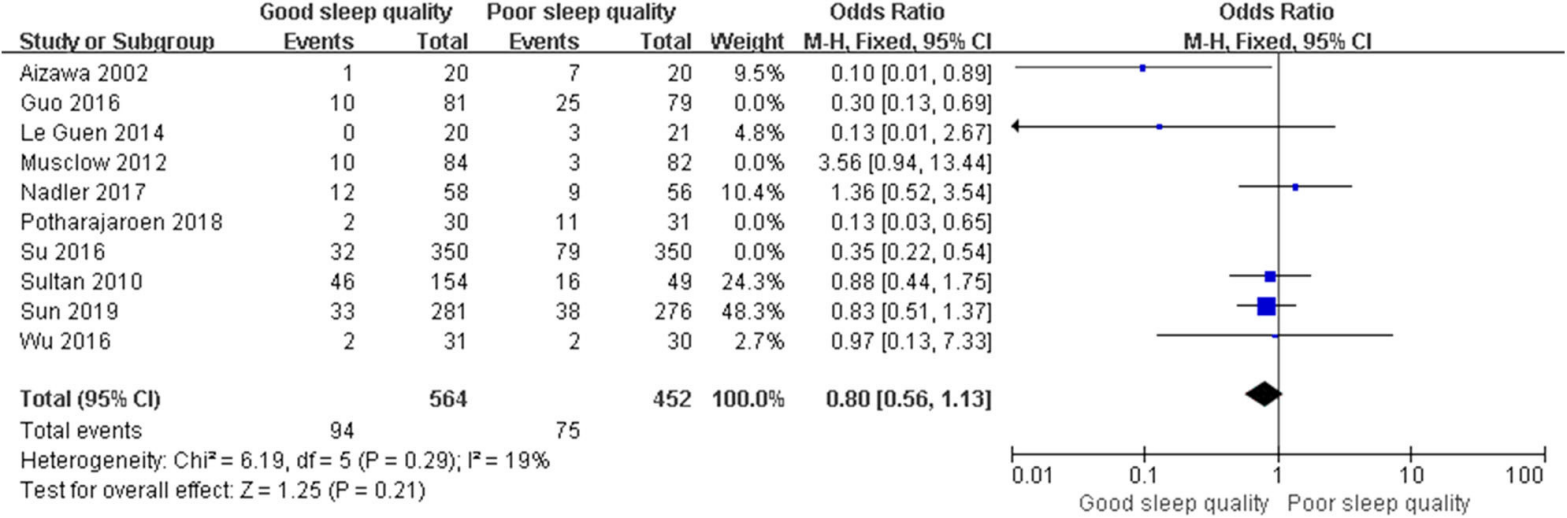
The pooled results of POD incidence after surgery between the patients with good and poor sleep quality in RCTs group after sensitivity analysis.

We conducted *post hoc* meta-analysis for the remaining literature in these groups using a fixed-effects model with OR, and the pooled results were consistent with those prior to sensitivity analysis (ROT group: OR = 0.65, 95% CI: [0.47, 0.91], *I*^2^ = 12%, *p* for effect = 0.01; RCT group: OR = 0.82, 95% CI: [0.52, 1.29], *I*^2^ = 19%, *p* for effect = 0.39) (Figures 6, 7).

## Discussion

This meta-analysis investigated the effect of perioperative sleep disturbance on the incidence of POD. The results from observational trials (retrospective and prospective) demonstrated that perioperative sleep disturbances were significantly associated with elevated POD incidence, while those from RCTs did not confirm this positive association.

POD, as a kind of mental disorder, is a knotty problem that patients may face following major surgery. It arises as the combined effect of multiple factors, which include advanced age, low education level, preoperative impaired cognition, alcohol abuse, smoking, cardiac or macrovascular surgery, major non-cardiac surgeries, perioperative administration of sedative and analgesic drugs, and postoperative imperfect analgesia, among others (53–55). Sleep problems are a hot topic in current clinical research due to their prevalence and potential negative impact on cognitive functions, including learning, memory, spatial orientation, behavioral capacity, and so on (9, 56–58). Furthermore, long-term sleep disturbances are intimately associated with major depression and dementia in adult populations, especially the aged (59, 60). The mechanism underlying sleep-disturbance-related cognitive dysfunction is still unclear. Some studies reported that poor sleep quality (OSA, disordered circadian rhythms, and psychologically-based sleep deprivation) could lead to neuronal apoptosis in those areas of the brain related to cognition, by means of neuroinflammation, changes in neurotransmitter activity (e.g., adenosine), and cerebral hypoxic and hypoperfusion injury (61–64). Other studies confirmed that sleep disturbance was rather common in the perioperative period, and that it seriously affected postoperative cognitive function (65, 66). A great number of studies have reported the effects of perioperative sleep disturbance on POD, and most of them concluded that poor perioperative sleep quality was an important risk factor of POD (67–69). We performed this meta-analysis to clarify and substantiate these findings, collecting as many articles as possible, listing their trial characteristics, and synthesizing their results.

All enrolled observational trials reported the POD incidence in patients exposed to sleep disturbances and non-sleep disturbances. Because the aim of this meta-analysis was to investigate the effect of perioperative sleep quality on POD, we selected studies with significant differences in sleep quality between intervention and control groups in RCTs. Different from the observational trials, in RCT group, some patients in the intervention group suffered sleep disturbances and some in the control group exhibited good sleep quality; thus, the negative pooled result of RCTs may be unreliable. We detected high heterogeneity in the ROT and RCT groups, which could affect the reliability of our meta-analysis results. We used sensitivity analysis—namely subgroup analysis and one-by-one literature exclusion—to address high heterogeneity (70, 71). In addition, though meta-analysis using a random-effect model did not solve heterogeneity, it did decrease the impact of significant heterogeneity on the pooled results (72). As a result, we conjectured that high heterogeneity may be the result of a combination of factors, and we used the method of one-by-one literature exclusion to solve this problem. Consequently, eight trials were excluded from the ROT and RCT groups (that is, 4 trials each). We then used a fixed-effects model with OR to conduct meta-analyses for the remaining literature in these groups, and the pooled results were consistent with those prior to sensitivity analysis.

Publication bias was another problem affecting the reliability of meta-analysis results (73). Detecting and adjusting publication bias is indispensable for meta-analysis. Currently, the primary methods for detecting publication bias include the rank correlation test (Begg's test, Schwarzer's test, and arcsine Begg's test), the regression test (Egger's test, Macaskill's regression, Harbord's test, Peters' test, and arcsine regression methods), and funnel plots. Funnel plots are not suitable for meta-analyses with few traits and high heterogeneity due to their tendency to produce asymmetric graphs (74, 75). In this meta-analysis, there were only seven trials in the ROT group, so we did not draw a funnel plot. Of the other methods, Egger's test has the highest power and the most accurate *p-*value, and it is easy to understand. As such, it is the most popular method for detecting publication bias (24). If the *p-*value for a given group was <0.05 following Egger's test, we determined that this group exhibited significant publication bias. We used trim-and-fill analysis to adjust publication bias (24). Unexpectedly, there was no evidence of significant publication bias in any of the three groups in this meta-analysis.

The advantages of this meta-analysis were as follows. First, this meta-analysis included as many trials as possible, and it did not exclude trials based on study methods. Second, we selected observational trials with exposure and non-exposure factors (sleep disturbances) and RCTs with significant differences in risk factors (sleep quality) between intervention and control groups; thus, the results were more convincing. Third, grouping the studies according to their different study methods helped us to synthesize data. Fourth, different sleep disturbances (OSA, disordered circadian rhythms, and psychologically-based sleep deprivation) were included in this meta-analysis, allowing us to analyze the effects of perioperative sleep quality on POD more comprehensively.

Several limitations should also be taken into consideration in our meta-analysis. First of all, although we observed a positive correlation between sleep disturbance and POD in the observational study groups (retrospective and prospective), this finding may be less reliable than it could be because of the inevitable selection bias (76). Meanwhile, the most enrolled observational studies in this meta-analysis presented small sample size, which may attenuate the reliability of synthesized results as well (77). Furthermore, the study from Gupta et al. (28) only provided the number of matched patients in the control group, therefore we were not sure whether real-world research would affect the pooled results. In addition, although there was a striking difference in sleep quality between the RCTs' intervention and control groups, some patients in the intervention group suffered sleep disturbances and some in the control group exhibited good sleep quality; thus, the negative pooled result of RCTs may be unreliable. Therefore, a real-world prospective observational study with large sample size can significantly elevate the validity and reliability of results in spite of selection bias (78, 79). Different onset time and POD assessment methods may also have affected the reliability of our pooled results. Lastly, the low-quality literature in each of the three groups likely compromised the reliability of our pooled results as well.

## Conclusion

This systematic review and meta-analysis demonstrated that perioperative sleep disturbances were significantly associated with elevated POD incidence in observational trials (retrospective and prospective), but not in RCTs. Despite this inconsistency, we suggest that perioperative sleep disturbance could be a potential risk factor for POD, and that clinicians should pay careful attention to this phenomenon. In the future, the high-quality real-world prospective observational trial with large sample size will be required to further prove the effect of perioperative sleep disturbances on POD.

## Data Availability Statement

The original contributions presented in the study are included in the article/Supplementary Material, further inquiries can be directed to the corresponding author/s.

## Author Contributions

HW and LZ were responsible for document retrieval and were responsible for adjusting data discrepancies. ZZ and YL performed the screening process for titles and abstracts. HW and SY performed the screening process for full texts. YL and QL were responsible for extracting the data. QL and SY independently assessed the quality of included studies. QL conducted the statistical analysis and made the figures and tables. HW prepared the manuscript. FY supervised the whole process and ensured the effectiveness of the meta-analysis. All authors read and approved the submission of the final manuscript.

## Conflict of Interest

The authors declare that the research was conducted in the absence of any commercial or financial relationships that could be construed as a potential conflict of interest.

## Supplementary Material

Supplementary Figure 1 Risk of bias graph: review authors' judgements about each risk of bias item presented as percentages across all included RCT study.

Supplementary Figure 2 Risk of bias summary: review authors' judgements about each risk of bias item for each included RCT study.

Supplementary Table 1 The guidelines of the 2009 PRISMA (Preferred Reporting Items for Systematic reviews and Meta-analyses).

Supplementary Table 2 Bias risk of observational studies (retrospective and prospective) by NOS.

Supplementary Table 3 Egger's test for publication bias of included trials.

Supplementary Table 4 Retrospective observational studies-meta regression based on risk factors of high heterogenicity.

Supplementary Table 5 RCT-meta regression based on risk factors of high heterogenicity.
