# Supplementary figures and images for: Perioperative Sleep Disturbances and Postoperative Delirium in Adult Patients: A Systematic Review and Meta-Analysis of Clinical Trials
Source: Front Psychiatry. 2020 Oct 14;11:570362. doi: 10.3389/fpsyt.2020.570362 (PMC7591683; doi:10.3389/fpsyt.2020.570362)

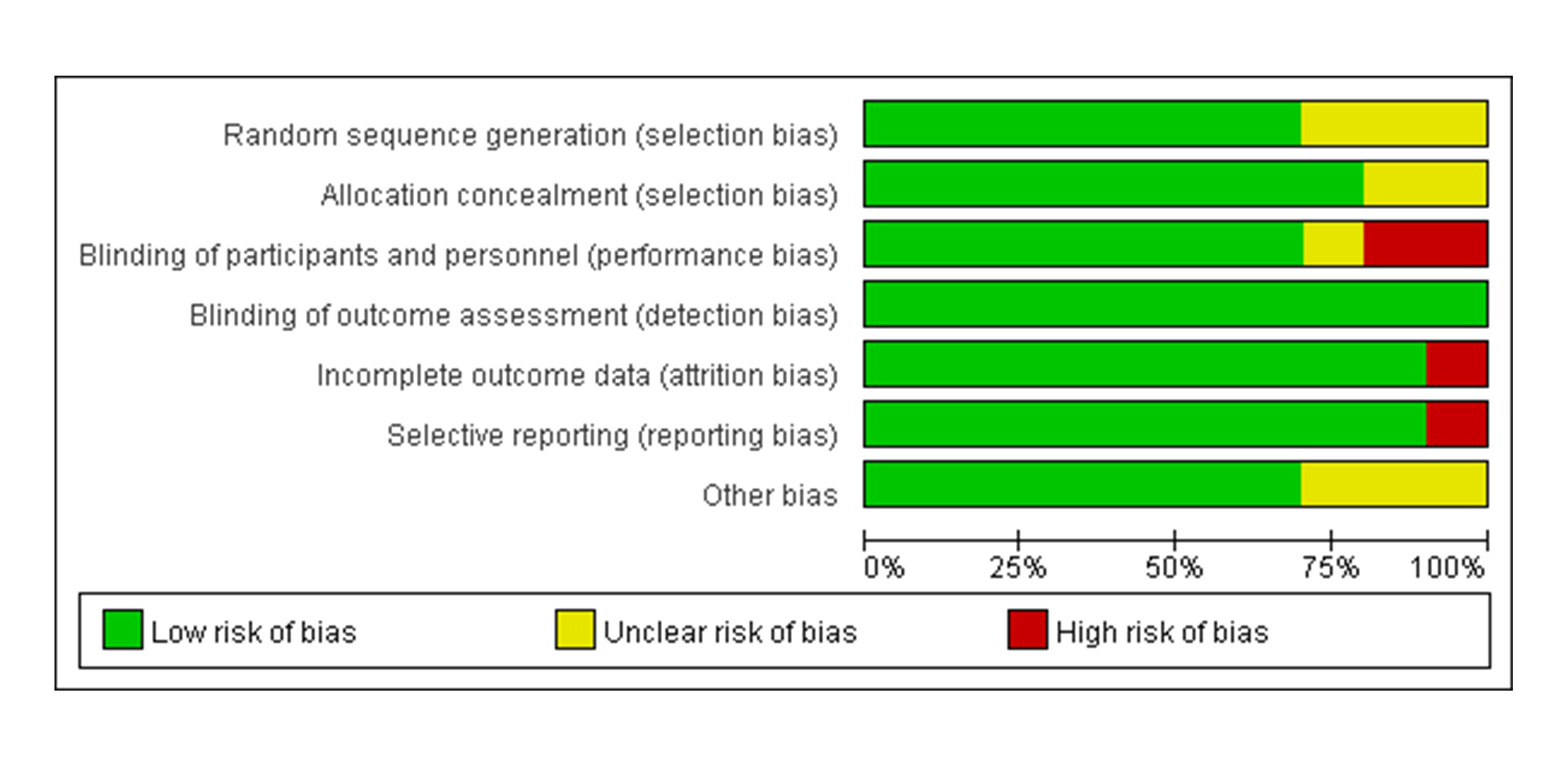

Supplement: Supplementary Figure 1 — Risk of bias graph: review authors' judgements about each risk of bias item presented as percentages across all included RCT study. [file Image_1.TIF]

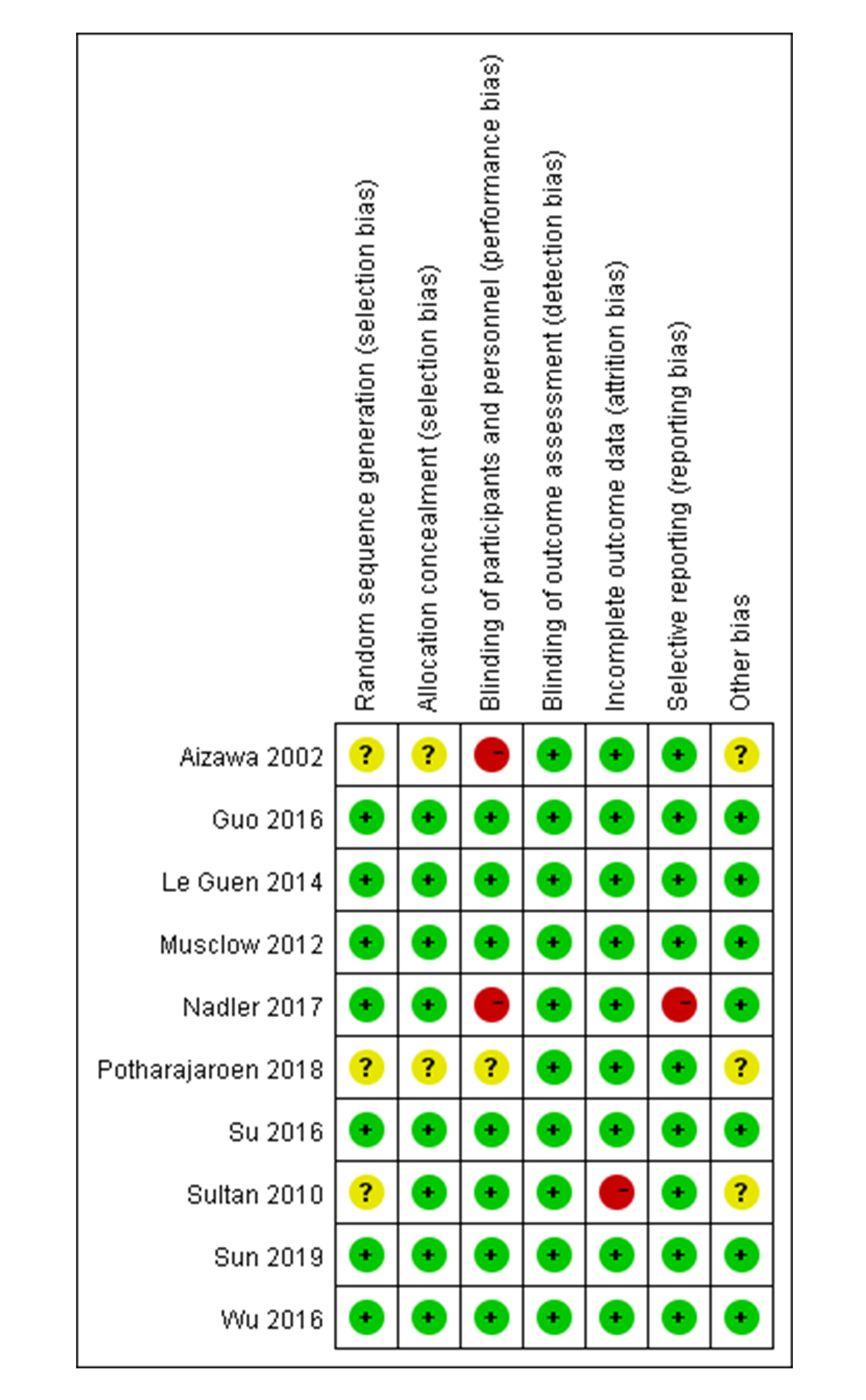

Supplement: Supplementary Figure 2 — Risk of bias summary: review authors' judgements about each risk of bias item for each included RCT study. [file Image_2.TIF]
