# Supplementary material for: Perioperative Sleep Disturbances and Postoperative Delirium in Adult Patients: A Systematic Review and Meta-Analysis of Clinical Trials
Source: Front Psychiatry. 2020 Oct 14;11:570362. doi: 10.3389/fpsyt.2020.570362 (PMC7591683; doi:10.3389/fpsyt.2020.570362)
Supplement: Supplementary Table 1 — The guidelines of the 2009 PRISMA (Preferred Reporting Items for Systematic reviews and Meta-analyses). [file Table_1.DOC]

| **Section/topic** | **#** | **Checklist item** | **Reported on page #** |
| --- | --- | --- | --- |
| **TITLE** | | |  |
| Title | 1 | Perioperative sleep disturbances and postoperative delirium in adult patients—a systematic review and meta-analysis of clinical trials | P 1 |
| **ABSTRACT** | | |  |
| Structured summary | 2 | The aim of this systematic review and meta-analysis of clinical trials was to investigate the effects of perioperative sleep disturbances on postoperative delirium (POD). The search and screening identified 29 trials including 55908 patients. Afterwards, we divided the included trials into 3 groups according to study designs: 7 retrospective observational trials, 12 prospective observational trials and 10 randomized controlled trials (RCTs) groups. The results demonstrated that perioperative sleep disturbances in observational groups were significantly associated with POD occurrence: retrospective [OR=0.56, 95% CI: (0.33, 0.93), I^2^=91%, *p* for effect =0.03] and prospective [OR=0.27, 95% CI: (0.20, 0.36), I^2^=25%, *p* for effect <0.001], whereas the incidence of POD was not significant difference between intervention and control groups in RCTs group [OR=0.58, 95% CI: (0.34, 1.01), I^2^=68%, *p* for effect =0.05]. The one-by-one literature exclusion method was used to solve high heterogeneity. Eventually, perioperative sleep disturbances were potential risk factors associated with POD by observational studies, but the positive result did not be obtained in RCTs group. | P 2 |
| **INTRODUCTION** | | |  |
| Rationale | 3 | According to reports, POD accounts for 11%-51% of patients after major surgery, and is independently associated with prolonged intensive care unit (ICU) and hospital stay, long-term postoperative cognitive dysfunction, and increased mortality. Perioperative sleep disturbances are common problems in surgery patients. Over 40% of patients complained poor sleep quality during the first night following surgery, and the sleep problems continued several days postoperatively. Some observational studies have reported that the patients with poor sleep quality were predisposed to mental disorders including delirium and cognitive dysfunction. In addition, several randomized controlled trials (RCTs) found that improvement of sleep quality through some medications or methods strikingly decreased the incidence of delirium as well | P 3 |
| Objectives | 4 | We designed this systematic review and meta-analysis to investigate the effect of sleep disturbances on incidence of delirium in patients undergoing surgery through synthesizing data of clinical trials. | P 3 |
| **METHODS** | | |  |
| Protocol and registration | 5 | No registration |  |
| Eligibility criteria | 6 | The inclusion criteria included: 1) participants with age≥18 yr; 2) the patients undergoing surgery; 3) the article reported the effect of sleep on delirium. The exclusion criteria were: 1) duplicate articles; 2) participants with age<18 years; 3) review or meta-analysis; 4) article published as abstract, letter, case report, basic research, editorial, note, method or protocol; 5) article presented in non-English language; 6) studies without statistical difference in sleep quality between intervention and control groups; 7) studies without specific number of patients with sleep problem (observational studies) and/or delirium; 8) studies including some patients without surgery. | P 4 |
| Information sources | 7 | We searched the databases including "Pubmed", "Embase", "Cochrane Library" and “Web of Science” through PICOS (Population, Intervention, Comparison, Outcome, Study design) method by the time to 12th May 2020. | P 3-4 |
| Search | 8 | The entry words included "sleep" OR "insomnia" OR "sleep disturbance" OR “night” OR “circadian” AND "surgery" OR "operation" OR "postoperative" OR "anaesthesia" OR "anesthesia" AND "delirium" OR “confusion” OR "agitation" OR “acute confusional state” OR “acute confusional syndrome” and the search scope was "title and abstract". Because all studies about effect of sleep disturbances on incidence of POD in adult patients undergoing surgery were eligible in this meta-analysis, we did not confine the search words of study designs. | P 4 |
| Study selection | 9 | The inclusion criteria included: 1) participants with age≥18 yr; 2) the patients undergoing surgery; 3) the article reported the effect of sleep on delirium. The exclusion criteria were: 1) duplicate articles; 2) participants with age<18 years; 3) review or meta-analysis; 4) article published as abstract, letter, case report, basic research, editorial, note, method or protocol; 5) article presented in non-English language; 6) studies without statistical difference in sleep quality between intervention and control groups; 7) studies without specific number of patients with sleep problem (observational studies) and/or delirium; 8) studies including some patients without surgery. | P 4 |
| Data collection process | 10 | Two authors were independently responsible for reviewing the titles, abstracts or both and summarized the data of the included literatures. Another two authors were in charge of the data discrepancy adjustment. | P 4-5 |
| Data items | 11 | 1) authors; 2) publication year; 3) number of the total participants in each study; 4) age range of all the participants; 5) country of publication; 6) percentage of males; 7) procedures that the participants underwent; 8) methods of sleep disturbance assessment; 9) methods of POD assessment; 10) number of patients with and without POD; 11) number of patients with good and poor sleep quality; 12) the follow-up time for delirium | P 4 |
| Risk of bias in individual studies | 12 | Two authors independently assessed the quality of included studies. The Cochrane Collaboration Risk of Bias Assessment tool was used to assess the risk of bias of all included RCTs, and the Newcastle-Otawa Quality Assessment Scale (NOS) was used to assess the bias risk of observational trials. If the two authors had the different assessment results, they consulted the third or the forth one. Eventually, we reached consensus. | P 5 |
| Summary measures | 13 | The dichotomous outcome were reported as odds ratios (OR) with 95% confidence interval (CI). The statistical tests were two-sided and *p* value for overall effect <0.05 was considered significant differences. | P 5-6 |
| Synthesis of results | 14 | The values of I^2^ and the Mantel-Haenszel chi-square test (*p* value for heterogeneity) were used to evaluate the heterogeneity of included studies. And the values of I^2^<40%, 40%-60%, and >60% represented low, moderate and high heterogeneity, respectively. A *p* value for heterogeneity<0.1 or I^2^ >50% was regarded as high heterogeneity and the method of random-effect model analysis was applied to pool the data. | P 5-6 |

Page 1 of 2

| **Section/topic** | **#** | **Checklist item** | **Reported on page #** |
| --- | --- | --- | --- |
| Risk of bias across studies | 15 | Bias risk of observational studies (retrospective and prospective) was assessed by NOS, and 15 trials were regarded as high quality as they obtained 7 stars or more respectively. Bias risk of 10 RCTs was assessed by the Cochrane Collaboration Risk of Bias Assessment tool. Random sequence generation was assessed as a low risk of bias in 7 studies (70%), allocation concealment was assessed in 8 studies (80%), blinding of participants was assessed in 7 studies (70%), blinding of outcome assessment was assessed in 10 studies (100%), incomplete outcome data was assessed in 9 studies (90%) and selective outcome reporting was assessed in 9 studies (90%). 6 RCTs were assessed to be high quality. | P7-8 |
| Additional analyses | 16 | Publication bias was assessed by Eegg’s test. A trim and fill analysis was performed to solve publication bias for the group with *p* value <0.05 by Eegg’s test. Meta-regression was performed to investigate the heterogeneity sources by assessing the potential factors including publication year, average age (≥ 65 years and < 65 years), male proportion (≥ 50% and < 50%), surgery types (non-cardiac surgery, cardiac surgery, and cardiac and non-cardiac surgeries), onset time for POD (> 3 d and ≤ 3 d), and study quality (low quality and high quality). All *P* values of these risk factors were over 0.05. | P6 |
| **RESULTS** | | |  |
| Study selection | 17 | See Fig. 1 | P 5-6 |
| Study characteristics | 18 | For each study, present characteristics for which data were extracted (e.g., study size, PICOS, follow-up period) and provide the citations. | P 6-7 |
| Risk of bias within studies | 19 | Present data on risk of bias of each study and, if available, any outcome level assessment (see item 12). | P 7-8 |
| Results of individual studies | 20 | For all outcomes considered (benefits or harms), present, for each study: (a) simple summary data for each intervention group (b) effect estimates and confidence intervals, ideally with a forest plot. | P 7-8 |
| Synthesis of results | 21 | The random-effect model with OR was selected due to high heterogeneity in ROTs group (I^2^=91%) and RCTs group (I^2^=68%), whereas the fixed-effect model with OR was selected because of low heterogeneity in POTs group (I^2^=25%). The pooled results demonstrated significant difference in incidence of POD after surgery between the patients with good and poor sleep quality in ROTs group [OR=0.56, 95% CI: (0.33, 0.93), I^2^=91%, *p* for effect =0.03] and in POTs group [OR=0.27, 95% CI: (0.20, 0.36), I^2^=25%, *p* for effect <0.001] (Fig 2 and 3). However, no significant difference was exhibited in group of RCTs [OR=0.58, 95% CI: (0.34, 1.01), I^2^=68%, *p* for effect =0.05] | P 8 |
| Risk of bias across studies | 22 | Present results of any assessment of risk of bias across studies (see Item 15). | P 6-7 |
| Additional analysis | 23 | Give results of additional analyses, if done (e.g., sensitivity or subgroup analyses, meta-regression [see Item 16]). | P 8-9 |
| **DISCUSSION** | | |  |
| Summary of evidence | 24 | This meta-analysis included 7 ROTs, 12 POTs and 10 RCTs to investigate the effect of perioperative sleep quality on incidence of POD. The results demonstrated that perioperative sleep disturbances was strikingly associated with the elevated incidence of POD in observational trials (retrospective and prospective), but the positive result did not be obtained in RCTs group. | P 9 |
| Limitations | 25 | First of all, although we obtained the positive result of meta-analysis in observational study groups (retrospective and prospective), the finding was less reliable because of the inevitable selection bias [78]. Furthermore, the study from Gupta et al. [28] only provided the number of matched patients in the control group, therefore we were not sure whether real-world research would affect the pooled results. In addition, although there was striking difference in the sleep quality between intervention and control groups of RCTs, some patients in intervention group suffered sleep disturbances and some in control group were in the state of good sleep quality, thus the negative pooled result of RCTs may be unreliable. Besides, different follow-up time and POD assessment methods possibly affected the reliability of pooled results. Lastly, the included low-quality literatures in each of the three groups likely led to unreliability of pooled results as well. | P 11-12 |
| Conclusions | 26 | This mea-analysis demonstrated that perioperative sleep disturbances were strikingly associated with the elevated incidence of POD in observational trials (retrospective and prospective), but the positive result did not be obtained in RCTs group. Despite inconsistent results obtained from the three groups, as potential risk factors, perioperative sleep disturbances should be highly paid attention to by clinicians. | P 12 |
| **FUNDING** | | |  |
| Funding | 27 | No. |  |

*From:*  Moher D, Liberati A, Tetzlaff J, Altman DG, The PRISMA Group (2009). Preferred Reporting Items for Systematic Reviews and Meta-Analyses: The PRISMA Statement. PLoS Med 6(7): e1000097. doi:10.1371/journal.pmed1000097

For more information, visit: **www.prisma-statement.org**.

Page 2 of 2
