# Supplementary material for: Perioperative Sleep Disturbances and Postoperative Delirium in Adult Patients: A Systematic Review and Meta-Analysis of Clinical Trials
Source: Front Psychiatry. 2020 Oct 14;11:570362. doi: 10.3389/fpsyt.2020.570362 (PMC7591683; doi:10.3389/fpsyt.2020.570362)
Supplement: Supplementary Table 2 — Bias risk of observational studies (retrospective and prospective) by NOS. [file Table_2.DOC]

| Study | Selection | | | | Comparability | Outcome | | | Total score |
| --- | --- | --- | --- | --- | --- | --- | --- | --- | --- |
|  | Representativeness of exposed cohort | Selection of unexposed  cohort | Ascertainment of exposure | Outcome of interest |  | Assessment of outcome | Follow-up long enough for outcomes to occur | Adequacy  of follow-up |  |
| Bosmak 2017 | * | * |  | * |  |  | * | * | 5 |
| Gupta 2001 | * | * | * |  | ** | * | * | * | 8 |
| He 2019 | * | * | * | * | * | * | * | * | 8 |
| King 2020 | * | * | * |  | ** | * | * | * | 8 |
| Pichler 2019 | * | * | * | * | * | * |  | * | 7 |
| Strutz 2019 | * | * | * |  | * | * | * | * | 8 |
| Wang 2015 | * | * |  | * | * | * | * | * | 7 |
| Cheraghi 2016 | * | * | * |  |  | * | * | * | 6 |
| Flink 2012 | * | * | * | * | * | * | * | * | 8 |
| Hwang 2018 | * | * | * | * | * | * | * | * | 8 |
| Koster 2009 | * | * |  | * | * | * | * | * | 7 |
| Roggenbach 2014 | * | * | * | * | * | * | * | * | 8 |
| Simeone 2018 | * | * |  | * | * | * | * |  | 6 |
| Tafelmeier 2019 | * | * | * | * | * | * | * | * | 8 |

Bias risk of observational studies (retrospective and prospective) by NOS

Continued

| Study | Selection | | | | Comparability | Outcome | | | Total score |
| --- | --- | --- | --- | --- | --- | --- | --- | --- | --- |
|  | Representativeness of exposed cohort | Selection of unexposed  cohort | Ascertainment of exposure | Outcome of interest |  | Assessment of outcome | Follow-up long enough for outcomes to occur | Adequacy  of follow-up |  |
| Todd 2017 | * | * | * | * | * | * | * | * | 8 |
| Wang 2018 | * | * | * | * | * | * | * | * | 8 |
| Wang 2020 | * | * | * | * | * | * | * | * | 8 |
| Yamagata 2005 | * | * |  | * | * |  | * | * | 6 |
| Zhang 2015 | * | * |  | * | * | * | * | * | 7 |
