# Supplementary material for: Perioperative Sleep Disturbances and Postoperative Delirium in Adult Patients: A Systematic Review and Meta-Analysis of Clinical Trials
Source: Front Psychiatry. 2020 Oct 14;11:570362. doi: 10.3389/fpsyt.2020.570362 (PMC7591683; doi:10.3389/fpsyt.2020.570362)
Supplement: Supplementary Table 3 — Egger's test for publication bias of included trials. [file Table_3.DOC]

Egger’s test for publication bias

|  | Std_Eff | Coef | Std. Err. | t | P>|t| | 95% Conf. Interval | |
| --- | --- | --- | --- | --- | --- | --- | --- |
| ROTs | slope | 0.2764296 | 0.170421 | 1.62 | 0.166 | -0.1616514 | 0.7145107 |
|  | bias | -3.05287 | 1.425809 | -2.14 | 0.085 | -6.718028 | 0.6122884 |
| POTs | slope | -1.393007 | 0.6431796 | -2.17 | 0.056 | -2.826101 | 0.040086 |
|  | bias | 0.1633052 | 1.212438 | 0.13 | 0.896 | -2.538175 | 2.864785 |
| RCTs | slope | -0.5302562 | 0.477877 | -1.11 | 0.299 | -1.632243 | 0.5717302 |
|  | bias | -0.1005055 | 1.160781 | -0.09 | 0.933 | -2.777271 | 2.576261 |
