# Supplementary material for: Perioperative Sleep Disturbances and Postoperative Delirium in Adult Patients: A Systematic Review and Meta-Analysis of Clinical Trials
Source: Front Psychiatry. 2020 Oct 14;11:570362. doi: 10.3389/fpsyt.2020.570362 (PMC7591683; doi:10.3389/fpsyt.2020.570362)
Supplement: Supplementary Table 4 — Retrospective observational studies-meta regression based on risk factors of high heterogenicity. [file Table_4.DOC]

Retrospective observational studies-meta regression based on risk factors of high heterogenicity

| _ES | Coef. | Std. Err. | t | P>|t| | 95% Conf. Interval | |
| --- | --- | --- | --- | --- | --- | --- |
| Publication year | 0.0378121 | 0.0415997 | 0.91 | 0.405 | -0.0691233 | 0.1447475 |
| Mean age (years) | 0.6521678 | 0.5316214 | 1.23 | 0.435 | -6.102723 | 7.407058 |
| Male (%) | -0.5643919 | 0.8233295 | -0.69 | 0.617 | -11.02579 | 9.897002 |
| Surgical types | 0.7691879 | 0.4933361 | 1.56 | 0.363 | -5.499242 | 7.037618 |
| Follow-up time | 1.137289 | 0.7423533 | 1.53 | 0.368 | -8.295204 | 10.56978 |
| Study quality | -1.430826 | 1.27932 | -1.12 | 0.464 | -17.68613 | 14.82448 |
