# Supplementary material for: Perioperative Sleep Disturbances and Postoperative Delirium in Adult Patients: A Systematic Review and Meta-Analysis of Clinical Trials
Source: Front Psychiatry. 2020 Oct 14;11:570362. doi: 10.3389/fpsyt.2020.570362 (PMC7591683; doi:10.3389/fpsyt.2020.570362)
Supplement: Supplementary Table 5 — RCT-meta regression based on risk factors of high heterogenicity. [file Table_5.DOC]

| _ES | Coef. | Std. Err. | t | P>|t| | 95% Conf. Interval | |
| --- | --- | --- | --- | --- | --- | --- |
| Publication year | -0.023793 | 0.079749 | -0.30 | 0.773 | -0.2076945 | 0.1601085 |
| Mean age (years) | -2.124613 | 1.719745 | -1.24 | 0.272 | -6.545358 | 2.296131 |
| Male (%) | -0.5743785 | 0.7804625 | -0.74 | 0.486 | -2.419879 | 1.271122 |
| Follow-up time | 0.4664084 | 0.6598276 | 0.71 | 0.500 | -1.055157 | 1.987974 |
| Study quality | 0.2501931 | 0.69038 | 0.36 | 0.726 | -1.341826 | 1.842212 |

RCT-meta regression based on risk factors of high heterogenicity
